# Supplementary material for: Real-Time Estimation of the Risk of Death from Novel Coronavirus (COVID-19) Infection: Inference Using Exported Cases
Source: J Clin Med. 2020 Feb 14;9(2):523. doi: 10.3390/jcm9020523 (PMC7074479; doi:10.3390/jcm9020523)
Supplement: Supplementary file 1 [file jcm-09-00523-s001.pdf]

Supplementary materials

A. Sensitivity analysis by varying the starting point of exponential growth of cumulative novel coronavirus incidence from 1 December to 10 December, 2019

The starting point of exponential growth of cumulative case incidence was fixed as the illness onset date of the index case (i.e, 8 December 2019) in Scenario 1, while it was allowed to vary from the day the first exportation case was observed (i.e., 13 January 2020) in Scenario 2. However, since there is uncertainty regarding the actual starting point of exponential growth in Scenario 1 due to discrepancies in the date of illness onset for the first reported case, a sensitivity analysis was conducted by varying this date from 1 December 2019 to 10 December 2019. Table S1 and Figure S1 show the estimated growth rate, cumulative incidence, and cCFR that result from this analysis.

Table S1. Sensitivity analysis with varying the start point of exponential growth in cumulative incidence from 1–10 December, 2019 in estimation Scenario 1.

The start point of exponential growth	Exponential growth rate (95% CI)	Estimated incidence in China on 24 January (95% CI)	cCFR on 24 January (95% CI)
1 December	0.12 (0.11, 0.12)	4718 (3328, 6278)	5.33% (3.50, 7.58)
2 December	0.12 (0.11, 0.13)	4896 (3473, 6472)	5.35% (3.57, 7.61)
3 December	0.12 (0.12, 0.13)	5087 (3584, 6733)	5.40% (3.59, 7.62)
4 December	0.13 (0.12, 0.13)	5392 (3730, 7024)	5.44% (3.62, 7.68)
5 December	0.13 (0.12, 0.14)	5506 (3908, 7309)	5.48% (3.66, 7.74)
6 December	0.14 (0.13, 0.14)	5733 (4067, 7583)	5.53% (3.68, 7.80)
7 December	0.14 (0.13, 0.15)	6089 (4317, 8099)	5.52% (3.68, 7.76)
8 December	0.14 (0.14, 0.15)	6266 (4435, 8285)	5.64% (3.74, 7.96)
9 December	0.15 (0.14, 0.16)	6565 (4663, 8687)	5.69% (3.79, 8.03)
10 December	0.15 (0.14, 0.15)	6924 (4885, 9211)	5.27% (3.51, 7.45)

cCFR, confirmed case fatality risk; CI, confidence interval (the 95% CI was derived from the Markov chain Monte Carlo method).

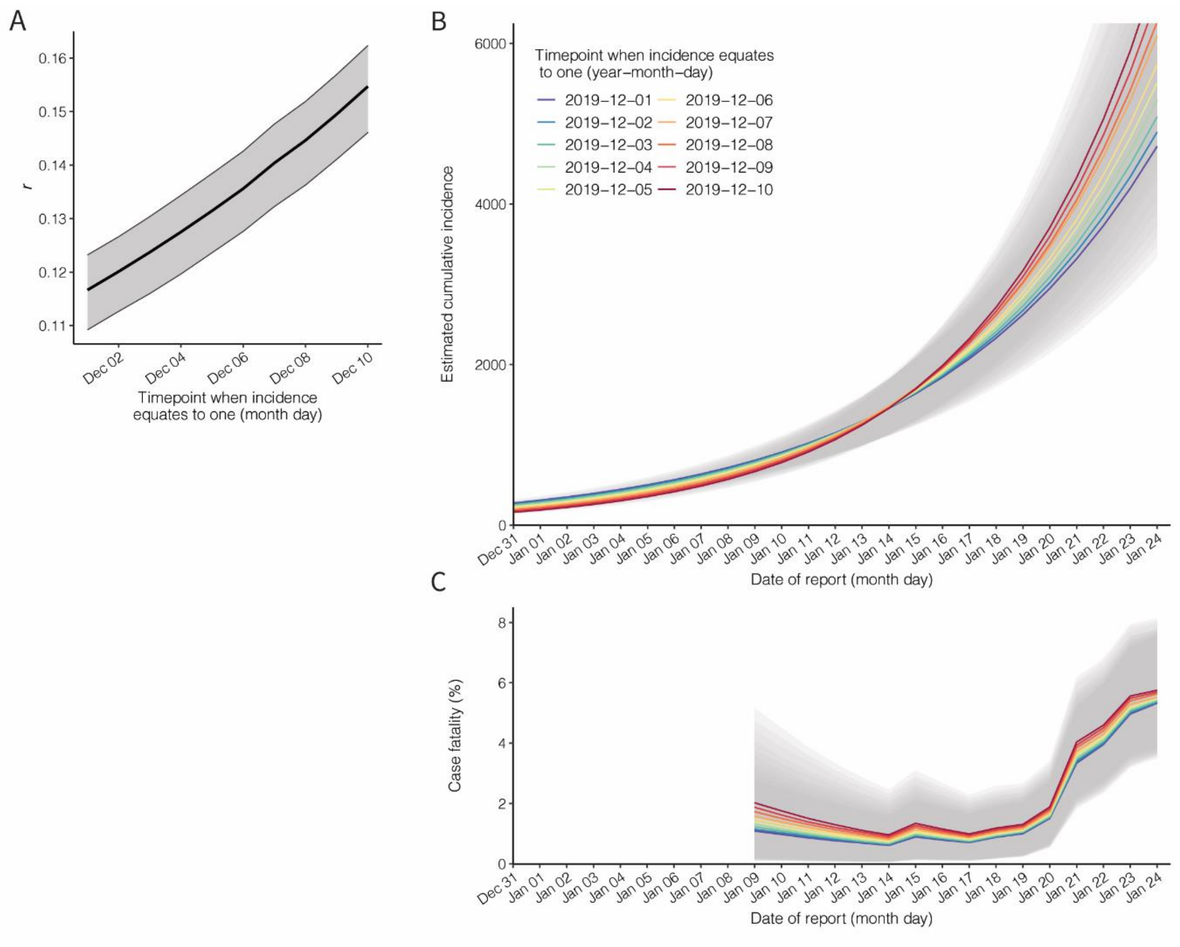

Figure S1. Sensitivity analysis with varied starting point for exponential growth of cumulative case incidence from 1–10 December, 2019. The estimated values of the: A) exponential growth rate, B) cumulative incidence and C) confirmed case fatality risk are shown by varying the start date of exponential growth in Scenario 1. Each line and shaded area present the estimate and its 95% confidence interval (derived from the Markov chain Monte Carlo method).

B. Sensitivity analyses by varying the data cutoff date from 15 January to 24 January, 2020

As the present study relies on real-time data, the estimates (i.e., growth rate, cumulative incidence, and cCFR) using different number data cutoff dates are shown in Figure S2 (Scenario 1) and S3 (Scenario 2).

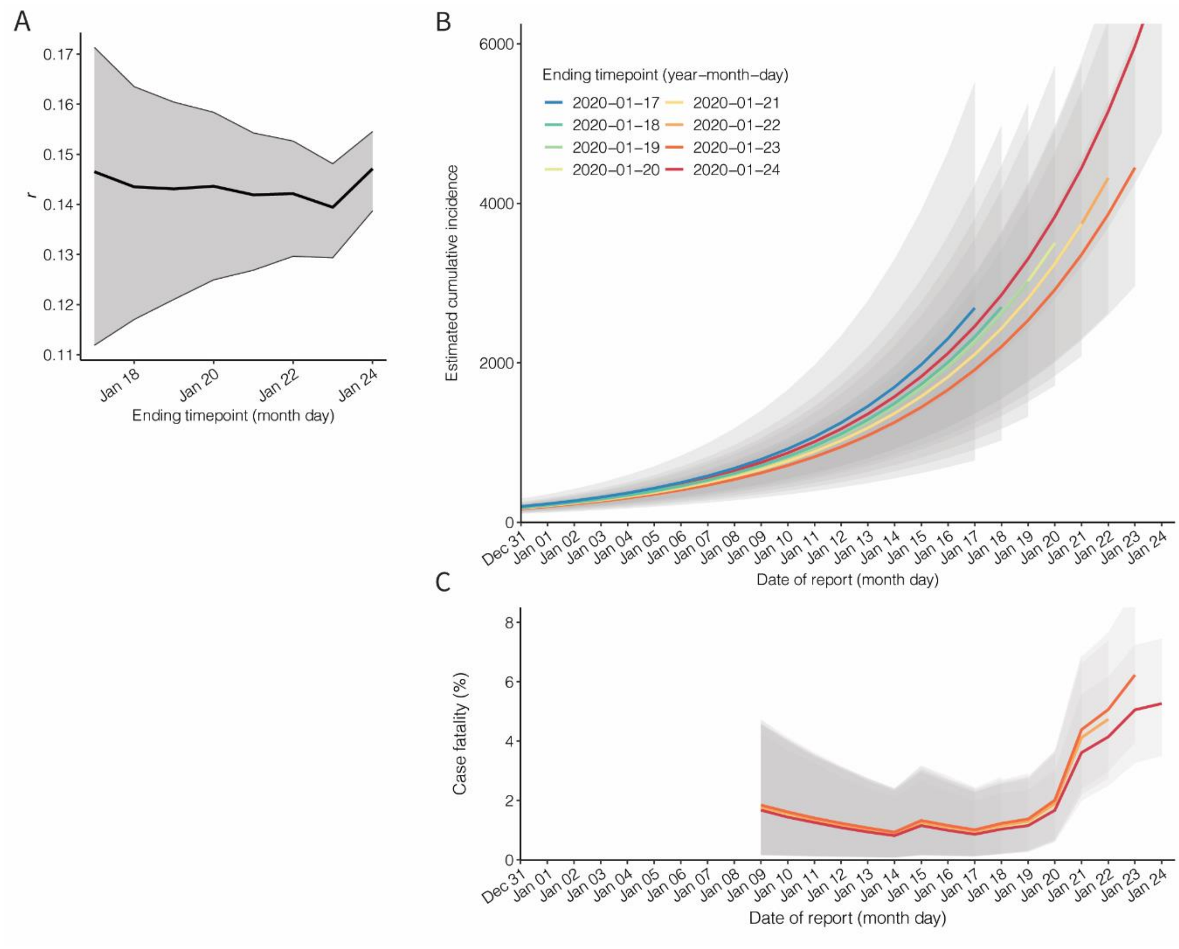

Figure S2. Sensitivity analysis in Scenario 1, varying the data cutoff date between 15 and 24 January 2020. The estimated values of the A) exponential growth rate, B) cumulative incidence, and C) confirmed case fatality risk depend on the data cutoff date (i.e., end point of each estimation). Each line and shaded area indicate the estimate and its 95% confidence interval.

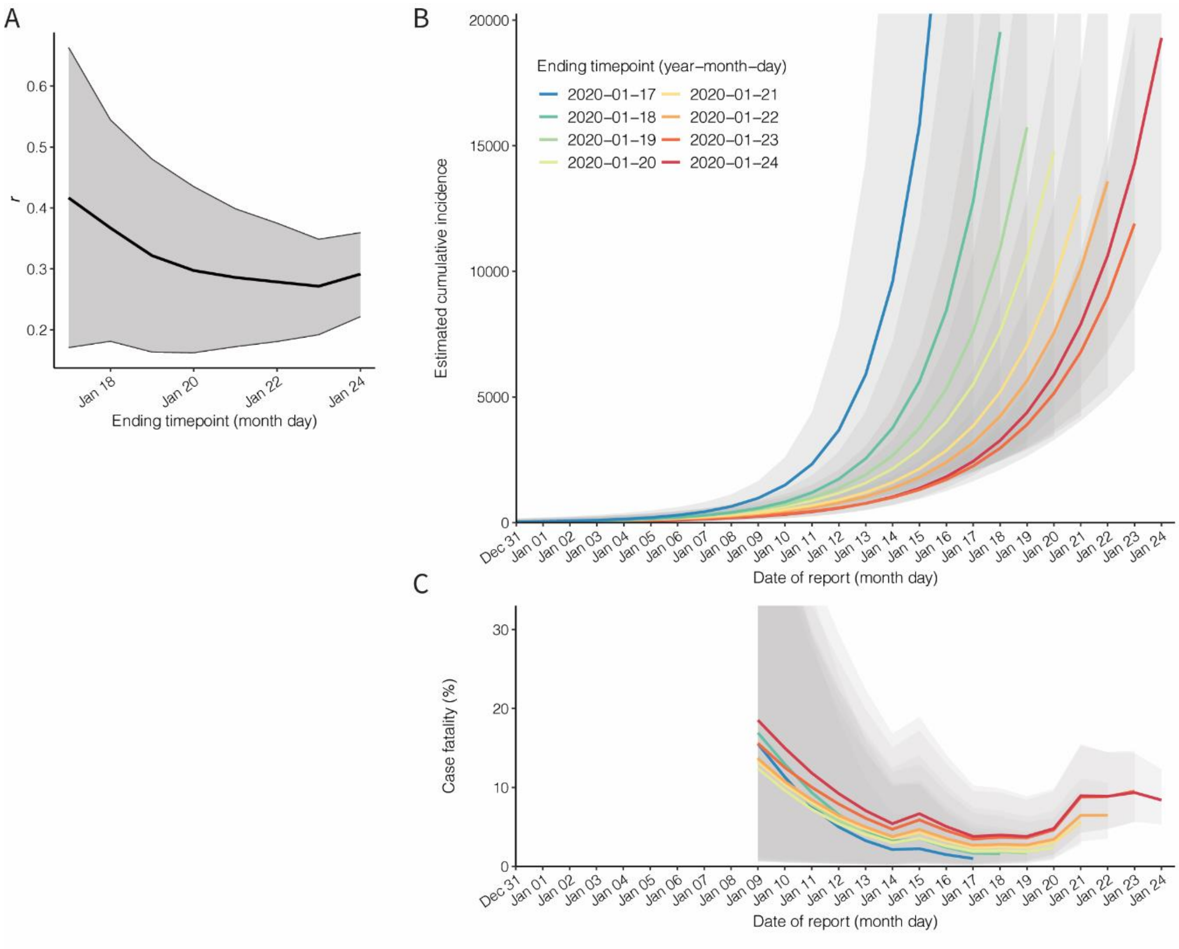

Figure S3. Sensitivity analysis in Scenario 2, varying the data cutoff date between 15 and 24 January 2020. The estimated values of the A) exponential growth rate, B) cumulative incidence, and C) confirmed case fatality risk depend on the data cutoff date (i.e., end point of each estimation). Each line and shaded area indicate the estimate and its 95% confidence interval.

C. Sensitivity analyses by varying the detection time window (*T*) and catchment population size in Wuhan airport (*n*)

In the present study, the detection time window was fixed at 12.5 days, based assumed values for the incubation and infectious periods. The catchment population in Wuhan airport was also fixed at 11 million. As part of the sensitivity analysis, Table S2 shows the estimated growth rate, cumulative incidence and cCFR by varying only detection time window, while Table S3 shows the estimates with different catchment population sizes and a fixed detection time window for Scenarios 1 and 2, respectively.

Table S2. Sensitivity analysis varying the detection time window (*T*) with a catchment population in Wuhan airport of 11 million

Scenario	Detection window time	Exponential growth rate (95% CI)	Estimated incidence on 24 January (95% CI)	cCFR on 24 January (95% CI)
1	3.6 days	0.18 (0.18, 0.19)	30094(21524, 39513)	1.82% (1.20, 2.58)
1	7.5 days	0.16 (0.15, 0.17)	12024 (8642, 15838)	3.54% (2.36, 4.99)
1	10 days	0.15 (0.14, 0.16)	8343 (5920, 11040)	4.61% (3.06, 6.48)
1	12.5 days	0.15 (0.14, 0.15)	6924 (4885, 9211)	5.27% (3.51, 7.45)
2	3.6 days	0.33 (0.25, 0.40)	80925 (45517, 127347)	2.77% (1.72, 4.14)
2	7.5 days	0.31 (0.23, 0.38)	34795 (19653, 54114)	5.31% (3.37, 7.87)
2	10 days	0.30 (0.23, 0.37)	26042 (14602, 41034)	6.64% (4.24, 9.82)
2	12.5 days	0.29 (0.22, 0.36)	19289 (10901, 30158)	8.39% (5.34, 12.26)

cCFR, confirmed case fatality risk; CI, confidence interval (the 95% CI was derived from Monte Carlo Markov Chain method).

Table S3. Sensitivity analysis by varying the catchment population size in Wuhan airport (*n*) with a fixed detection time window of 12.5 days

Scenario	Catchment population in Wuhan airport	Exponential growth rate (95% CI)	Estimated incidence on 24 January (95% CI)	cCFR on 24 January (95% CI)
1	11 million	0.15 (0.14, 0.15)	6924 (4885, 9211)	5.27% (3.51, 7.45)
1	15 million	0.15 (0.15, 0.16)	9225 (6546, 12187)	4.29% (2.85, 6.04)
1	19 million	0.16 (0.15, 0.17)	12463 (8892, 16416)	3.46% (2.30, 4.88)
2	11 million	0.29 (0.22, 0.36)	19289 (10901, 30158)	8.39% (5.34, 12.26)
2	15 million	0.30 (0.23, 0.37)	27494 (15549, 42771)	6.41% (4.06, 9.49)
2	19 million	0.31 (0.23, 0.38)	36005 (20348, 56273)	5.19% (3.27, 7.68)

cCFR, confirmed case fatality risk; CI, confidence interval (the 95% CI was derived from the Markov chain Monte Carlo method).
